# Prognostic Significance of Stromal Periostin Expression in Non-Small Cell Lung Cancer

**DOI:** 10.3390/ijms21197025

**Published:** 2020-09-24

**Authors:** Katarzyna Ratajczak-Wielgomas, Alicja Kmiecik, Jedrzej Grzegrzołka, Aleksandra Piotrowska, Agnieszka Gomulkiewicz, Aleksandra Partynska, Konrad Pawelczyk, Katarzyna Nowinska, Marzenna Podhorska-Okolow, Piotr Dziegiel

**Affiliations:** 1 Division of Histology and Embryology, Department of Human Morphology and Embryology, Wroclaw Medical University, 50-368 Wroclaw, Poland; alicja.kmiecik@umed.wroc.pl (A.K.); jedrzej.grzegrzolka@umed.wroc.pl (J.G.); aleksandra.piotrowska@umed.wroc.pl (A.P.); agnieszka.gomulkiewicz@umed.wroc.pl (A.G.); aleksandra.partynska@student.umed.wroc.pl (A.P.); katarzyna.nowinska@umed.wroc.pl (K.N.); piotr.dziegiel@umed.wroc.pl (P.D.); 2 Department of Thoracic Surgery, Wroclaw Medical University, 53-439 Wroclaw, Poland; kopaw@wp.pl; 3 Department of Thoracic Surgery, Lower Silesian Centre of Lung Diseases, 53-439 Wroclaw, Poland; 4 Division of Ultrastructure Research, Wroclaw Medical University, 50-368 Wroclaw, Poland; marzenna.podhorska-okolow@umed.wroc.pl; 5 Department of Physiotherapy, University School of Physical Education, 51-612 Wroclaw, Poland

**Keywords:** periostin, non-small cell lung carcinoma, cancer-associated fibroblasts (CAFs), tumour microenvironment, cancer

## Abstract

Background: The microenvironment of solid tumours is significant in cancer development and progression. The aim of this study was to determine periostin (POSTN) expression by cancer-associated fibroblasts (CAFs) in non-small-cell lung cancer (NSCLC), as well as to assess associations with clinicopathological factors and prognosis. Materials and Methods: Immunohistochemical analysis of POSTN expression was performed on NSCLC (*N* = 700) and non-malignant lung tissue (NMLT) (*N* = 110) using tissue microarrays. Laser capture microdissection (LCM) for isolation of stromal and cancer cells of NSCLC was employed, and subsequently, POSTN mRNA expression was detected by real-time PCR. Immunofluorescence reaction and colocalisation analysis were performed by confocal microscopy. Results: Expression of POSTN in CAFs was significantly higher in NSCLC and in the adenocarcinoma (AC) and squamous cell carcinoma (SCC) subtypes compared to NMLT. POSTN expression in CAFs increased with clinical cancer stage, grades (G) of malignancy, and lymph node involvement in NSCLC. Higher POSTN expression in CAFs was an independent prognostic factor for overall survival (OS). LCM confirmed significantly higher POSTN mRNA expression in the stromal cells (CAFs) compared to the lung cancer cells. Conclusions: POSTN produced by CAFs might be crucial for NSCLC progression and can be an independent negative prognostic factor in NSCLC.

## 1. Introduction

Lung cancer remains the leading cancer worldwide with regard to incidence and mortality rates [1,2]. Most lung cancers are classified as non-small-cell lung cancers (NSCLCs—85%) or small-cell lung cancers (SCLC—15%) [1,2,3]. While there are rare types of NSCLCs, the three most prevalent histological types include adenocarcinomas (ACs), squamous cell carcinomas (SCCs), and large-cell carcinomas (LCCs) [3,4]. SCCs are believed to arise from metaplastic cells in the large airways, and they are most commonly associated with smoking, while ACs are malignant cancers that form glands and papillary structures. They are the most common histological type in non-smokers [3,4].

The majority of patients are diagnosed at an advanced tumour stage and therefore are not candidates for curative surgical resection. These patients receive multimodal chemotherapy, with or without radiotherapy [5,6]. Although the prognosis for NSCLC has improved, new prognostic markers and therapeutic strategies are still needed [7].

Most NSCLCs are characterised by fibrous connective tissue proliferation. The stromal changes are accompanied by fibroblast differentiation into cancer-associated fibroblasts (CAFs), which alter the extracellular matrix (ECM) components in tumour areas and become a potent supporter of carcinogenesis, promoting the initiation of epithelial tumour formation, angiogenesis, and metastasis [8,9]. Therefore, both the development and progression of NSCLCs are considered to be complex processes in which the tumour microenvironment plays an important role [10]. The tumour microenvironment is comprised of complicated dynamic microenvironments which are formed during tumour development and metastasis. It consists of non-tumour cells (i.e., CAFs, endothelial cells, or infiltrating leukocytes) and a large list of extracellular matrix (ECM) proteins and soluble components such as hormones, growth factors, or cytokines. Tumour cells are able to alter the microenvironment, and the composition of the tumour microenvironment influences the biological behaviours of carcinoma cells [11,12]. In consequence, these interactions between cancer cells and different components of the tumour microenvironment are currently considered essential in the process that leads to cancer transformation [12]. Hence, it is believed that understanding this mutual relationship would eventually enable us to treat cancer patients by targeting CAFs.

POSTN is an extracellular matrix N-glycoprotein with known functions in bone development, maturation and repair, tissue repair, and epithelial–mesenchymal transition (EMT) [13,14,15]. POSTN has been shown to regulate cancer cell proliferation, angiogenesis, invasion, and metastasis by interacting with integrins such as αVβ1, αVβ3, αVβ5, and α6β4, which activate the Akt/PKB and FAK-mediated signalling pathways [16,17,18,19]. Furthermore, POSTN acts as an important extracellular adhesion molecule to mediate communication between the cells and their extracellular microenvironments. Current studies have also demonstrated that POSTN plays pivotal roles in establishing and remodelling various tumour microenvironments [19,20]. Although the stroma of cancer tissues is the main source of POSTN, it is still unclear how POSTN facilitates the interplay between cancer cells and CAFs in NSCLC, thereby promoting tumour initiation and progression by modifying the tumour microenvironment.

Growing evidence has implicated that POSTN is overexpressed in various types of human cancer tissues, including ovarian cancer [21], breast cancer [22,23], colon cancer [24], head and neck cancer [25], and pancreatic ductal adenocarcinoma [26]. However, the effect of POSTN expression in CAFs on the progression of NSCLC remains largely unknown despite some published reports related to NSCLC [6,7,27]. In addition, the expression and function of POSTN in stromal cells (CAFs) in NSCLC have not been sufficiently studied, especially in respect to two distinctive histological groups, i.e., squamous cell cancer (SCC) and adenocarcinoma (AC). Furthermore, to the best of our knowledge, this is the first study with such an expanded cohort to analyse POSTN expression in CAFs in NSCLC. In addition, the results of our studies tackle for the first time the subject of POSTN expression on the mRNA level in the microdissected stromal cells (CAFs) compared to cancer cells and non-malignant lung cells.

In view of the above facts, the aim of this study was to determine the localisation of POSTN and the level of its expression in NSCLC, as well as to compare it with the expression level of commonly used diagnostic markers such as p63 and thyroid transcription factor 1 (TTF-1) proteins, which are routinely used to distinguish morphological subtypes of NSCLCs [28]. Moreover, we analysed the relationship between POSTN expression in stromal cells (CAFs) and clinicopathological factors to determine its prognostic and predictive value on a large and homogeneous group of patients.

## 2. Results

### 2.1. Immunohistochemical POSTN Expression in NSCLC

**Figure 1:**
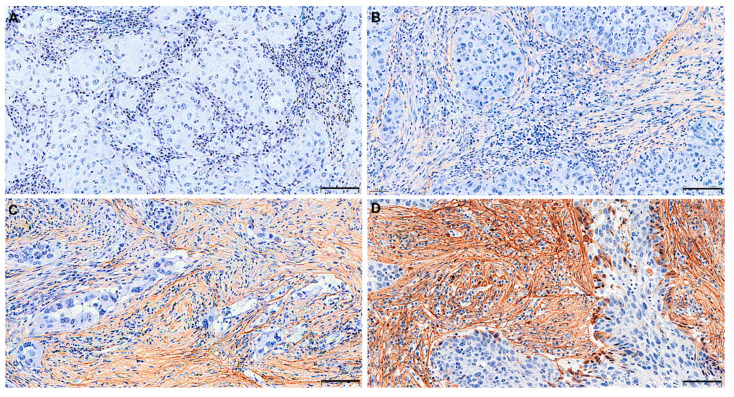
Representative immunohistochemical images with stromal periostin (POSTN) expression in non-small cell lung carcinoma (NSCLC), scored as (**A**) 0—no reaction, (**B**) 1—weak expression, (**C**) 2—moderate expression, and (**D**) 3—strong expression. Magnification 200×. Bar = 100 µm.

The expression of POSTN was noted mainly in tumour stromal cells of NSCLC and, in some cases, in tumour cells). Therefore, in this study, an attempt was made to evaluate the intensity of POSTN expression only in stromal cells (Figure 1A–D).

**Figure 2:**
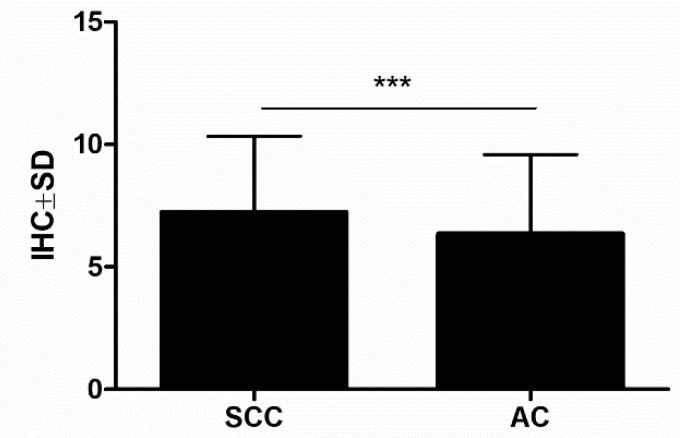
Expression levels of periostin (POSTN) in the tissue of two non-small cell lung carcinoma (NSCLC) subtypes: squamous cell cancer (SCC) and adenocarcinoma (AC) (*** *p* < 0.001); Mann–Whitney *U* test.

POSTN stromal cell expression was noted in 695 out of 700 (99.3%) NSCLC samples. The mean value of POSTN expression was 6.80 ± 2.50. Based on statistical analysis, sections scored 0–7 points were regarded as “low”, whereas sections scored 8–12 points were regarded as “high”. High POSTN expression in CAFs cells was noted in 334 (47.7%) of the analysed NSCLC cases, whereas low POSTN expression in stromal cells (CAFs) was detected in 366 (52.3%) of the NSCLC cases. Furthermore, POSTN expression in stromal cells was significantly higher in the SCC (7.22 ± 3.11) compared to the AC subtype (6.34 ± 3.24) (Figure 2).

**Figure 3:**
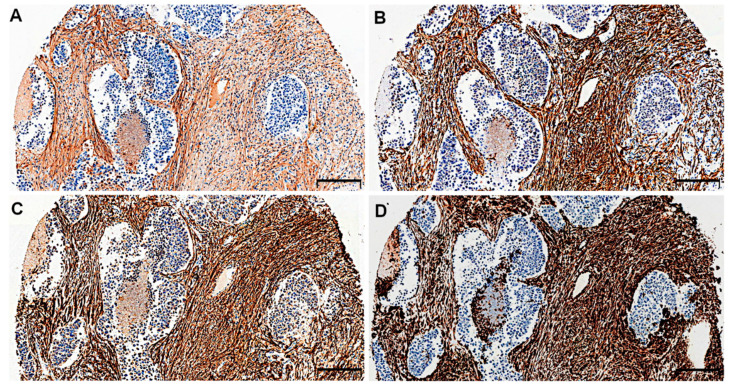
Serial sections of non-small cell lung carcinoma (NSCLC) immunostained for (**A**) periostin (POSTN), (**B**) α-smooth muscle actin (α-SMA), (**C**) podoplanin (D2-40), and (**D**) vimentin in cancer-associated fibroblasts (CAFs). Magnification 50×. Bar = 200 µm.

POSTN expression in tumour stromal cells (i.e., CAFs) was evidenced on serial sections of tissues by the positive IHC reaction for α-smooth muscle actin (α-SMA), podoplanin (D2–40), and vimentin, which are characteristic markers of CAFs. The IHC staining demonstrated the similar pattern of expression of these markers in NSCLC tumour stroma (Figure 3A–D).

### 2.2. Relationship between Immunohistochemical POSTN Expression in NSCLC and the Clinicopathological Data of Patients

**Figure 4:**
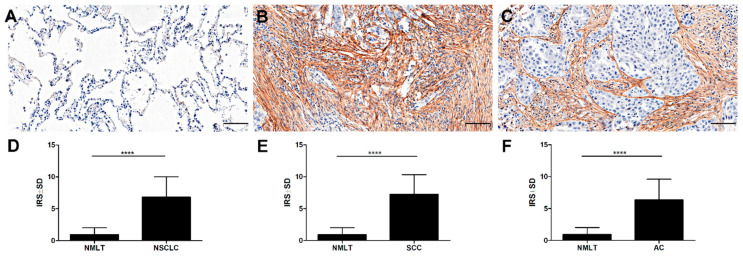
Expression of periostin (POSTN) in (**A**) non-malignant lung tissue (NMLT), (**B**) squamous cell cancer (SCC), and (**C**) adenocarcinoma (AC). Immunohistochemical evaluation of periostin (POSTN) expression level in (**D**) non-small cell lung carcinoma (NSCLC), (**E**) squamous cell cancer (SCC), and (**F**) adenocarcinoma (AC) compared to non-malignant lung tissue (NMLT), (**** *p* < 0.0001); Mann–Whitney *U* test. Magnification 200×. Bar = 100 µm.

A significantly higher level of POSTN expression in CAFs was observed in NSCLC as well as in the SCC and AC subtypes than in non-malignant lung tissue (NMLT) (**** *p* < 0.0001, respectively; Mann–Whitney *U* test); (Figure 4A–F).

**Figure 5:**
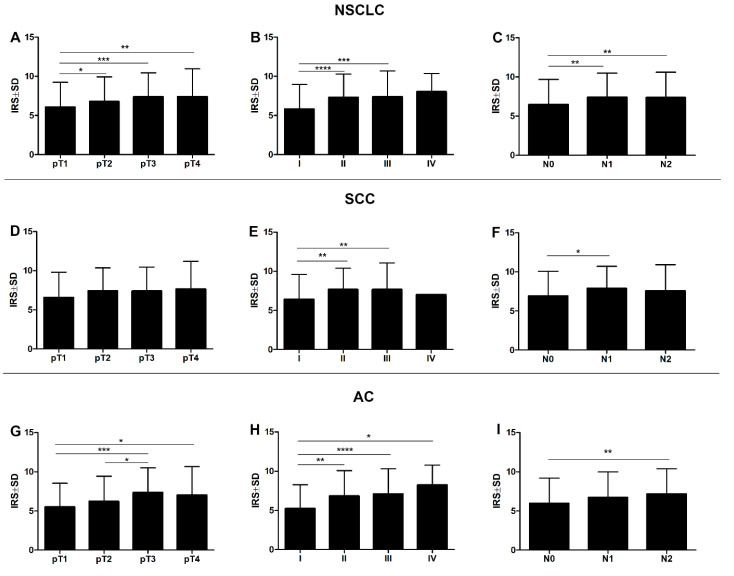
Expression level of periostin (POSTN) with regard to the clinicopathological factors of patients. Comparison of immunohistochemical periostin (POSTN) expression in the stromal cells (CAFs) of the non-small cell lung carcinoma (NSCLC), squamous cell cancer (SCC), and adenocarcinoma (AC) subtypes, (**A**,**D**,**G**, respectively) according to the tumour size (* *p* < 0.05, ** *p* < 0.005, *** *p* < 0.001), (**B**,**E**,**H**) clinical cancer stage (* *p* < 0.05, ** *p* < 0.005, *** *p* < 0.001, **** *p* < 0.0001), and (**C**,**F**,**I**) lymph node status (* *p* < 0.05, ** *p* < 0.005); Mann–Whitney *U* test.

Additionally, a significantly higher expression level of POSTN in CAFs was noted in pT3-pT4 as compared to pT1 tumours in the whole cohort of patients and in the AC cases (*** *p* < 0.001, ** *p* < 0.005, * *p* < 0.05, respectively; Mann–Whitney *U* test), (Figure 5A,G). A significant difference was also noted between pT1 and pT2 in the whole study cohort (* *p* < 0.05, Mann–Whitney *U* test). However, in the SCC group, no statistically significant differences were shown in POSTN expression in CAFs in relation to pT (Figure 5D). The level of this expression increased with an increasing clinical cancer stage in the whole study cohort (**** *p* < 0.0001, *** *p* < 0.001), as well as in the particular histological types, i.e., SCC and AC. A significant difference was noted between stages I and II, and I and III (** *p* < 0.005, **** *p* < 0.0001, respectively; Mann–Whitney *U* test), (Figure 5B,E,H). Statistically significant differences were also seen between stages I and IV in the AC group (* *p* < 0.05, Mann–Whitney *U* test).

Significant differences in POSTN expression in CAFs were also noted in relation to the lymph node status. POSTN expression in CAFs was significantly higher in the group of patients with lymph node metastasis (N2) than the one with N0 in the whole study cohort and the AC group (** *p* < 0.005 in both cases, Mann–Whitney *U* test), (Figure 5C,I). Moreover, NSCLC tissues and SCC cases with metastases to hilar and intrapulmonary lymph nodes (N1) displayed higher expression of POSTN in CAFs compared to the group of patients without lymph node metastases (N0) (** *p* < 0.005, * *p* < 0.05 Mann–Whitney *U* test), (Figure 5C,F).

**Figure 6:**
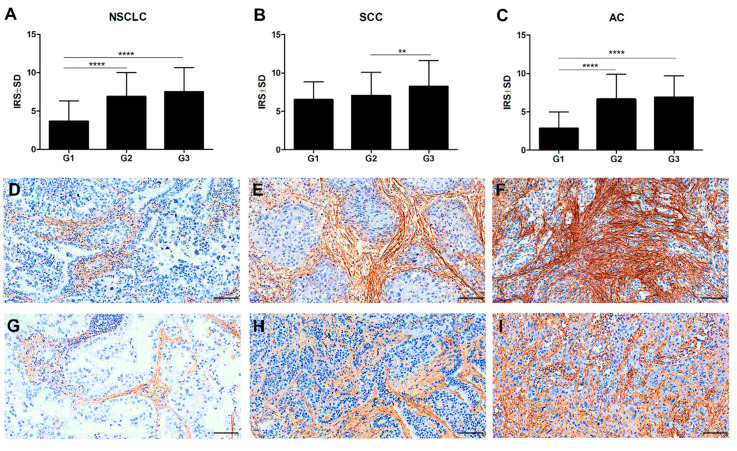
Comparison of periostin (POSTN) expression levels in different grades of malignancy (**G**) of the tumour in (**A**) non-small cell lung carcinoma (NSCLC) (**** *p* < 0.0001), (**B**) squamous cell cancer (SCC) (** *p* < 0.005), and (**C**) adenocarcinoma (AC) (**** *p* < 0.0001); Mann–Whitney *U* test. Immunohistochemical expression of periostin (POSTN) in stromal cells increased with a higher malignancy grade (**G**) in squamous cell cancer (SCC): G1 (**D**), G2 (**E**), G3 (**F**), and in adenocarcinoma (AC): G1 (**G**), G2 (**H**), and G3 (**I**). Magnification 200×. Bar = 100 µm.

In addition, the Mann–Whitney *U* test demonstrated an increasing level of POSTN expression in CAFs as the malignancy grade (G) of the tumour increased. Significant differences were noted between G1 tumours and those of G2 and G3 in the whole study cohort (**** *p* < 0.0001 in both cases), (Figure 6A), as well as in the AC cases (**** *p* < 0.0001 in both cases, Mann–Whitney *U* test), (Figure 6C,G–I). Additionally, significant differences were found between G2 and G3 tumours in the SCC cases (** *p* < 0.005, Mann–Whitney *U* test), (Figure 6B,D–F).

### 2.3. Associations between POSTN and TTF-1, p63, and D2-40 Expression Levels and Cancer Cell Proliferation

The correlation analysis showed a low positive significant correlation of POSTN in CAFs with p63 (*r* = 0.11, ** *p* < 0.005) in NSCLC. Moreover, in the whole study group, POSTN expression in CAFs revealed a low negative significant correlation with TTF-1 (*r* = −0.23, *** *p* < 0.001).

**Figure 7:**
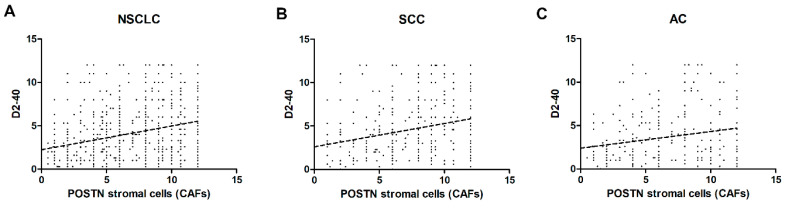
Correlations of periostin (POSTN) expression levels in stromal cells (CAFs) with podoplanin (D2-40) in (**A**) non-small cell lung carcinoma (NSCLC) (*r* = 0.27, *** *p* < 0.001), (**B**) squamous cell cancer (SCC) (*r* = 0.25, *** *p* < 0.001), and (**C**) adenocarcinoma (AC), (*r* = 0.20, *** *p* < 0.001).

We also noticed a weakly positive correlation of POSTN expression by CAFs with D2-40 in stromal cells in the whole cohort of patients as well as in the SCC and AC subtypes (*r* = 0.27, *** *p* < 0.001, *r* = 0.25, *** *p* < 0.001, *r* = 0.2, *** *p* < 0.001, respectively) (Figure 7A–C).

Additionally, POSTN expression in stromal cells of NSCLC correlated weakly positively with the expression of the Ki-67 antigen (*r* = 0.14, * *p* < 0.05).

### 2.4. Prognostic Significance of POSTN Expression in NSCLC

**Figure 8:**
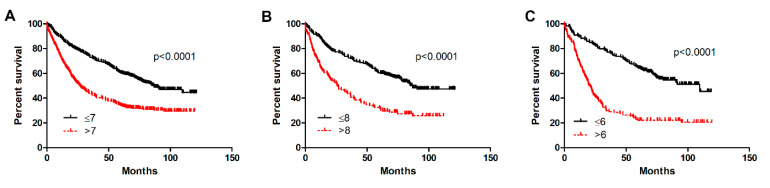
Kaplan–Meier survival curves reflecting the prognostic impact of periostin (POSTN) expression by cancer-associated fibroblasts (CAFs) on overall survival (OS) of patients with non-small cell lung cancer (NSCLC). The prognostic impact of periostin (POSTN) expression levels (immunohistochemistry, IHC) was studied in (**A**) the whole patient cohort (**** *p* < 0.0001) and in individual histological types, i.e., (**B**) squamous cell cancer (SCC) (**** *p* < 0.0001) and (**C**) adenocarcinoma (AC) (**** *p* < 0.0001); Mann–Whitney *U* test. Cut-off points for the analysis were estimated based on the median.

The prognostic impact of POSTN expression in NSCLC was analysed with respect to the overall survival (OS) of patients independently in the whole study cohort as well as in AC and SCC. The log-rank (Mantel–Cox) analysis showed that a higher expression of POSTN in CAFs in NSCLC was related to OS. Patients with high POSTN expression in CAFs had a significantly shorter survival compared to the group with low expression in the whole study group (**** *p* < 0.0001), as well as in the two particular histological subtypes, i.e., AC and SCC (**** *p* < 0.0001, **** *p* < 0.0001, respectively), (Figure 8A–C).

Univariate analysis demonstrated that some clinicopathological factors were significantly associated with a poorer OS. From among the factors considered in this group, a higher POSTN expression in CAFs, age ≥ 63 years, a larger primary tumour size (T2–T4), lymph node metastasis, an advanced stage (II–IV) (in the whole study cohort and in AC and SCC), the smoking status, and a high expression of Ki-67 (AC) were associated with a poor prognosis (Table 1A,B).

Multivariate survival analysis was performed for all factors that were significantly associated with OS in the univariate analysis. The Cox proportional hazards regression model revealed that POSTN in CAFs was an independent prognostic indicator of OS in patients with NSCLC in the whole study cohort as well as in the AC and SCC subtypes (Table 1A,B). The multivariate analysis also demonstrated that tumour size, the presence of lymph node metastases, and age were independent prognostic factors in the whole study cohort and in the AC cases (Table 1A,B). Moreover, in the SCC subgroup, tumour size and age were found to be an independent prognostic marker of OS (Table 1B).

### 2.5. Immunofluorescence

Double immunofluorescence staining for α-SMA and POSTN in NSCLC showed that α-SMA+ fibroblasts were embedded in cancer stroma, which contained abundant immunoreactive POSTN. Focal colocalisation of POSTN and α-SMA signals occurred (*r* = 0.37).

**Figure 9:**
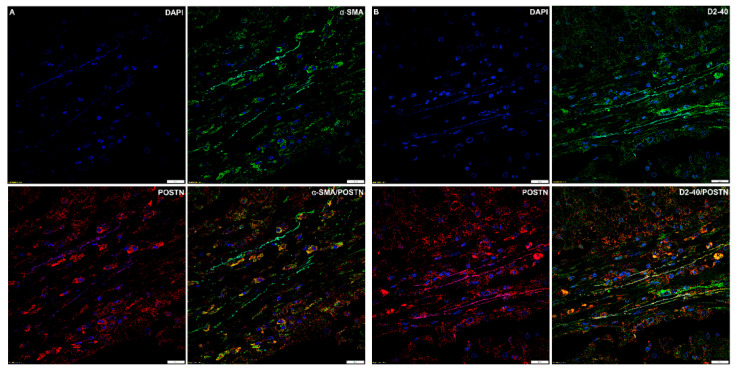
Confocal images showing colocalisation of (**A**) periostin (POSTN)/α-smooth muscle actin (α-SMA) (*r* = 0.37) and (**B**) periostin (POSTN)/podoplanin (D2-40) (*r* = 0.28) in non-small cell lung carcinoma (NSCLC).. Objective 60×/1.40 Oil; pinhole airy 1.25. Bar = 20 µm.

Moreover, double immunofluorescence staining for POSTN and D2-40 showed focal colocalisation of D2-40+ cells with POSTN immunoreactivity (*r* = 0.28) (Figure 9A,B).

### 2.6. Laser Capture Microdissection and Real-Time PCR

**Figure 10:**
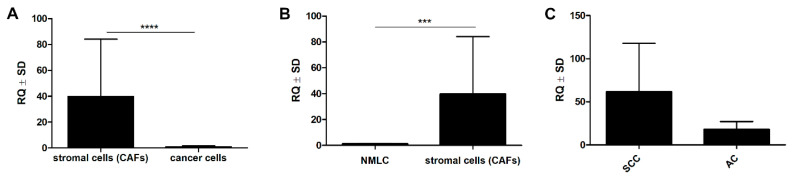
Significantly higher mRNA expression of periostin (POSTN) in the microdissected (LCM) stromal cells (CAFs) as compared to their expression in (**A**) cancer cells (**** *p* < 0.0001) and (**B**) non-malignant lung cells (NMLC) (*** *p* < 0.001). (**C**) Comparison of mRNA periostin (POSTN) expression in squamous cell cancer (SCC) and its expression in adenocarcinoma (AC); Mann–Whitney *U* test.

Significantly higher mRNA expression of POSTN was observed in the microdissected stromal cells (CAFs) compared to lung cancer cells (**** *p* < 0.0001, Mann–Whitney *U* test) (Figure 10A). Additionally, in the material from LCM, a significant difference in POSTN mRNA level was noted between the CAF cells and the microdissected non-malignant lung cells (NMLC), (*** *p* < 0.001, Mann–Whitney *U* test), (Figure 10B). POSTN mRNA expression in CAFs was also higher in the SCC subgroup compared to the AC cases (Figure 10C).

## 3. Discussion

Recent studies have shown that targeting the tumour microenvironment, which has been identified as one of the driving factors of tumour progression and invasion, could be an effective strategy for cancer treatment [29]. Fibroblasts are of particular interest inside this microenvironment. They can be activated into CAFs and consequently contribute to tumour growth, invasion, and metastasis [8]. Due to the close relationship between cancer cells and CAFs, it is increasingly clear that the development of cancer cannot be dissociated from its local microenvironment. The phenotype of a tumour is considered to be largely determined by the interactions between the cancer cells and their microenvironment. Therefore, the analysis of the cancerous stroma is of pivotal importance to better understand cancer [30].

To date, little has been known about the role of the tumour microenvironment (and particularly CAFs) in the development and progression of NSCLC. The present study has been the first to include such an expanded cohort with the aim to analyse POSTN expression in the stromal compartment of NSCLC and to try to determine the clinical and prognostic relevance of POSTN-positive CAFs in NSCLC. The source of POSTN in tumour is still under discussion. Recently, more and more research studies have indicated that POSTN is a matrix-specific protein with high expression in the stromal cells surrounding the carcinoma epithelium [30,31,32]. At the same time, some researchers have suggested that POSTN can be detected in cancer cells [26]. In our study, we found that the POSTN protein was mainly located in the stromal compartment of the tumour. We also observed that the pattern of the localisation of POSTN in the tumour stroma appears typically fibrillar and branched, suggesting a possible association between POSTN and the fibres of the desmoplastic stroma of NSCLC, which is in line with Hong et al. [27]. In addition, we were able to show that POSTN expression was predominantly localised in tumour stroma cells, i.e., in CAFs, as evidenced by the positive IHC staining and a very similar pattern in the serial sections for POSTN and CAF-associated markers, i.e., α-SMA, D2-40, and vimentin. These results may suggest that stromal cells, including CAFs, could be the main source of POSTN. The aforementioned observations were confirmed by laser confocal microscopy. We were able to demonstrate the existence of a focal colocalisation between POSTN and CAF-associated markers, i.e., α-SMA and D2-40. This clearly indicates that CAFs are stromal cells that express POSTN. We believe no previous studies have shown a positive significant correlation of POSTN in tumour stromal cells with D2-40 (CAFs) in NSCLC as well as in the AC and SCC subtypes, a correlation that may suggest the association of POSTN with CAF cells in tumour stroma. Kikuchi et al. [33] showed that fibroblast immunoreactive for α-SMA appeared in the cancer stroma, and immunoreactive POSTN was observed around these cells. This suggests that POSTN is expressed by CAFs. In their later studies, Kikuchi et al. [34] confirmed that α-SMA-positive CAFs are the primary source of POSTN, which facilitates tumour cell invasion by inducing EMT and establishing a neoplastic niche in gastric cancers. They also confirmed a focal colocalisation of POSTN and α-SMA, which we also did. The authors also found that POSTN-expressing CAFs constituted a cancer-promoting microenvironment [34], which is also in line with the tendency observed in our studies. Similarly, Underwood et al. [35] provided evidence of a potential mechanism by showing that the invasion-promoting effect of CAFs was partly modulated by POSTN. Furthermore, they also showed that CAFs isolated from oesophageal adenocarcinoma (EAC) had a functional myofibroblastic phenotype and promoted tumour cell invasion in vitro and growth in vivo [35]. An interesting conclusion was also drawn by Ryner et al. [36], who showed that POSTN was a key component of the reactive stroma in ovarian cancer and highlighted the important interplay between cancer and the tumour microenvironment. Additionally, Choi et al. [37] showed that POSTN was expressed in CAFs in human epithelial ovarian cancer (EOC), which was also consistent with our results. These authors also demonstrated that POSTN’s immunoreactivity was significantly observed in α-SMA-positive cells and also detected in the stroma surrounding α-SMA-positive CAFs, suggesting that CAFs were responsible for the deposition of POSTN in the stroma. Furthermore, Qin et al. [38] showed that the tumour stroma (especially CAFs) was an important source of POSTN in head and neck cancer tissue, and that the fibroblast-secreted POSTN created a tumour-supportive microenvironment to facilitate the growth and metastasis of head and neck cancer cells, a tendency we could also observe.

For this study, we also assessed the prognostic value of POSTN-positive CAFs in patients with NSCLC. The lowest value of POSTN was found in non-malignant lesions. However, it was significantly higher in NSCLC and in two individual histological types: SCC and AC. These results are consistent with the studies conducted by Murakami et al. [10] and Hong et al. [27], which may suggest a role of POSTN secreted by CAFs in the process of cancerous transformation in NSCLC. We also observed a positive correlation between the expression of POSTN and p63, which is a marker used to differentiate SCC from other NSCLC subtypes. Moreover, we demonstrated a negative correlation between the expression of POSTN in CAFs and TTF-1, which is commonly expressed by lung ACs. The association of POSTN expression with p63 and a negative correlation with TTF-1 in all the studied cases could be related to POSTN overexpression in the SCC subtype. It is important to mention that the relationship between the expressions of the above proteins has not been studied yet. The present paper also shows that POSTN-positive CAFs increased with the histological grade (G) of NSCLC and achieved their highest value in G3 and their lowest in G1 tumours, both in the whole cohort and in the particular histological subtypes (AC and SCC). Similar results were reported in NSCLC by Nitsche et al. [6] and Murakami et al. [10], although they included a significantly smaller pool of lung cancer cases and no SCC or AC cases. Furthermore, as observed in other cancers [30,34,35,39], an increased expression of POSTN was also significantly associated with clinical stage, regional lymph node (N) metastasis, and the primary tumour (T) stage, suggesting that POSTN secreted by CAFs could be a key element in the progression of NSCLC. On the other hand, it was reported that a decreased expression of POSTN was associated with the progression of bladder cancer in humans [40]. These results differed from our findings, which showed upregulation of POSTN in NSCLC. We speculate that there are two explanations for this. Firstly, it is possible that POSTN may function differently by expressing alternative splicing events at the C-terminal region, as eight different spliced transcripts of POSTN are produced [41]. Secondly, POSTN may have different functions according to different histopathological types of cancer.

The results we obtained with the use of the LCM method demonstrated the expression of mRNA POSTN in the microdissected CAFs, which suggests that stromal cells (particularly CAFs) could be the main source of POSTN in NSCLC. In addition, we noticed significantly upregulated POSTN mRNA in CAFs compared to lung cancer cells and NMLC. We also showed increased expression of POSTN mRNA in the SCC subtype compared to AC, which confirms the results of our IHC analysis. Li et al. [30] also used the LCM method to screen stroma-associated proteins involved in nasopharyngeal carcinoma (NPC) carcinogenesis. These authors confirmed a significantly higher POSTN expression in NPC stroma compared to normal nasopharyngeal mucosa (NNM) stroma. They suggested that POSTN was a potential biomarker for the differentiation and prognosis of NPC, and that it might play a critical role in NPC progression.

The analysis of the OS of NSCLC patients showed a worse prognosis in cases with higher POSTN expression in CAFs compared to patients with low expression of the protein, indicating the influence of this glycoprotein in cancer development. Our studies are in line with the results obtained by Okazaki et al. [7], Hong et al. [27], Soltermann et al. [32], and Takanami et al. [42], who also indicated that increased POSTN expression was associated with a shorter survival of patients with NSCLC. Our results were also in agreement with the previously established fact that the primary, important prognostic factors in patients with NSCLC are disease stagings (TNM, pT, and pN) [6,10]. It should be emphasized that our research has been the first to show a relationship between high POSTN expression in CAFs and a significantly shorter OS on a large population of patients with NSCLC as well as in two histological subtypes (AC and SCC). The Cox regression test showed that POSTN protein was detected as an independent prognostic factor, which had previously been observed by Hong et al. [27] and Murakami et al. [10]. However, these results are in contrast with the findings obtained by Takanami et al. [42]. These differences may be due to the different methodologies of research or to the use of different patient pool sizes.

In summary, the results of this study confirmed that POSTN was up-regulated in the stromal compartment (CAFs) of NSCLC compared to the control lung tissue, suggesting that POSTN may be related to the process of carcinogenesis in NSCLC. Additionally, our research demonstrated an increased expression of this glycoprotein in cases with higher pT, pN, clinical cancer stage, and histological grade (G) of the tumour. These findings highlight the importance of the tumour microenvironment (particularly POSTN-positive CAFs) in tumour progression in lung cancer. Additional multivariate analysis indicated that POSTN in CAF cells was an independent negative prognostic marker in the whole study cohort as well as in two histological subtypes of NSCLC (SCC and AC). Therefore, POSTN may be regarded as a potentially attractive therapeutic intervention target for lung cancer therapy.

These results may suggest that CAFs may play a key role in the development of NSCLC, which may eventually allow us to treat cancer patients by targeting CAF-positive POSTN.

Our study is not free from several limitations. Although it was conducted on one of the largest cohorts, it is a single institutional study and the results still need to be validated in a multicenter group of patients.

## 4. Materials and Methods

### 4.1. Patient Cohort

The study was performed on 700 paraffin-embedded tissue samples from patients diagnosed with NSCLC (including 370 SCCs and 330 ACs), as well as 110 paraffin-embedded samples of non-malignant lung tissue (NMLT). Patients with NSCLC underwent major resection of the lung parenchyma or sublobar resection at the Department of Thoracic Surgery of the Wroclaw Medical University, Poland. The histopathological evaluation of haematoxylin and eosin (H&E) stained slides was used to determine the type and the malignancy grade of the tumours (G) according to the World Health Organization criteria, and the pathological staging was standardized according to the 8th TNM edition [43]. Laser microdissection was performed on 10 frozen NSCLC fragments (5 ACs and 5 SCCs) and 6 NMLTs as the control.

The experiment was performed in accordance with the ethical standards and the approval of the Bioethics Committee of the Wroclaw Medical University (decision no. KB-100/2020, February 11th, 2020). All patients provided a written informed consent for the use of the material samples for scientific research. The correlations between POSTN expression by CAFs and the clinicopathological characteristics of patients with NSCLC are presented in Table 2.

### 4.2. Construction of Tissue Microarrays (TMA)

The lung TMA was constructed with archival formalin-fixed, paraffin-embedded lung tissue samples. The histological slides stained with H&E were prepared and scanned with the use of the Pannoramic Midi II histological scanner (3D HISTECH Ltd., Budapest, Hungary). Three representative cancer sites were selected by the Pannoramic Viewer (3D HISTECH Ltd., RRID: SCR_014424) Software. Subsequently, microarray samples were punched out from the selected regions of each donor block and transferred with a core of 1.5 mm into a new recipient paraffin block using the TMA Grand Master (3D HISTECH Ltd.).

### 4.3. TMA Immunohistochemistry (IHC)

TMA blocks were cut into 4-µm sections. IHC reactions were performed using the Dako Autostainer Link48 (Dako, Glostrup, Denmark). Deparaffinisation, rehydration, and epitope retrieval (97°C, 20 min) were performed using a low pH Target Retrieval Solution (Dako/Agilent Technologies, Santa Clara, CA, USA) in a PT- Link (Dako). Subsequently, the sections were washed in Tris-buffered saline and incubated with primary antibodies at room temperature for 20 min. The following specific primary antibodies were used: polyclonal rabbit anti-Periostin (dilution 1:200; code no.NBP1-82472; Novus Biologicals, Littleton, CO, USA), monoclonal mouse anti-Ki-67 antibody (ready-to-use, Clone MIB-1, code IS626; Dako), anti-TTF-1 (ready-to-use, Clone 8G7G3/1, code IR056; Dako), anti-p63 (ready-to-use, Clone DAK-p63, code IR662; Dako), anti-Podoplanin (ready-to-use, clone D2-40 (PDPN), code ISO072; Dako), anti-Vimentin (ready-to-use, clone V9, code GA630; Dako), and anti-αSMA (ready-to-use, clone IS611, code 1A4; Dako). The sections were then visualized using an EnVision FLEX kit (Dako). All slides were counterstained with haematoxylin (Dako). Negative control sections were generated in the absence of the primary antibody.

### 4.4. Evaluation of IHC Reactions

The evaluation of individual histological slides was conducted by two independent pathologists (P.D.; K.N.) using the BX-41 light microscope (Olympus, Tokyo, Japan, RRID:SCR_017022). Controversial cases were discussed until consensus was achieved. POSTN expression in CAFs was assessed using the immunoreactive score (IRS) of Remmele and Stegner [44]. This scale evaluates (A) the percentage of cells with a noticeable reaction (0 points, no cells with positive reaction; 1 point, 1–10% cells with positive reaction; 2 points, 11–50%; 3 points, 51–80%; 4 points, over 80% cells) and (B) the intensity of the colour reaction (0 points, no reaction; 1 point, low intensity; 2 points, moderate intensity; 3 points, strong colour reaction). The final score represents the product of the two values and falls in the range of 0–12 (AxB). The expression levels of nuclear proteins (TTF-1, p63, and Ki-67) were evaluated using a semi-quantitative five-grade scale based on the proportion of cells with the reaction product (0 points, no reaction; 1 point, 1–10%; 2 points, 11–25%; 3 points, 26–50%; 4 points, over 50% of cells had the reaction product) [45].

### 4.5. Laser Capture Microdissection (LCM)

Frozen tissue samples from 10 NSCLC (5 SCCs, 5 ACs) and 6 NMLT cases were used for LCM. Tissue sections (8 µm) were cut on a Leica CM1950 cryostat (Leica Microsytems, Wetzlar, Germany) and placed on a PET membrane slide (MMI, Glattbrugg, Switzerland). The slides were then fixed in 100% isopropyl alcohol and stained using the H&E staining kit Plus for LCM (MMI). LCM was performed using the MMI CellCut Plus system (MMI). The dissected samples were collected on the adhesive lids of 500 µL tubes (MMI). Total RNA from 10 microdissected stromal and cancer cells of NSCLC and 6 NMLT was isolated with the use of an RNeasy Micro kit (Qiagen, Hilden, Germany), according to the manufacturer’s instructions. A QuantiTect Reverse Transcription kit (Qiagen, Hilden, Germany) was used for cDNA synthesis, and finally real-time PCR reactions were performed.

### 4.6. Real-Time PCR

The reactions were performed in triplicates and evaluated by real-time PCR using the 7500 Fast Real-Time PCR System (Applied Biosystems, Foster City, CA, USA, RRID:SCR_014596), primers, and probes of the TaqMan system (Applied Biosystems). Hs00170815_m1 for POSTN and Hs99999903_m1 for ACTB (Applied Biosystem) were the primers and TaqMan probes that were used in the study. All reactions were performed under the following conditions: activation of polymerase at 50°C for 2 min, initial denaturation at 94°C for 10 min and 40 cycles of denaturation at 94°C for 15 s, followed by annealing and elongation at 60°C for 1 min. The results were standardised in relation to the expression of the reference gene of β-actin. The relative expression of POSTN mRNA (RQ) was calculated with the ΔΔ*C*_t_ method.

### 4.7. Confocal Microscopy

For immunofluorescence (IF), deparaffinisation, and hydration, thermal epitope demasking was done using a low pH Target Retrieval Solution (Agilent Technologies) for 20 min at 97°C in a Dako PT Link (Dako) apparatus. Sites of non-specific binding were blocked using 3% BSA in PBS (1 h/RT). The slices were incubated at 4°C overnight with primary anti-POSTN antibodies (dilution 1:200; code no. NBP1-82472; Novus Biologicals) in 3% BSA/PBS, anti-Podoplanin (ready-to-use, clone D2-40 (PDPN), code ISO072; Dako), and anti-αSMA (ready-to-use, clone IS611, code 1A4; Dako). Next, the preparations were incubated for 1 h with donkey anti-rabbit secondary Alexa Fluor 568 conjugated antibody (dilution 1:2000, Abcam, Cambridge, UK, Cat#ab175470, RRID:AB_2783823) and anti-mouse Alexa Fluor 488 conjugated antibody (dilution 1:2000, Abcam, Cambridge, UK, Cat# ab150113, RRID:AB_2756499).

Negative controls were performed with 1% BSA in PBS instead of the specific antibody. The preparations were mounted in a Prolong DAPI Mounting Medium (Invitrogen, Carlsbad, CA, USA). The observations were made at objective 60×/1.40 oil; with the use of a Fluoview FV3000 confocal microscopy (Olympus, RRID:SCR_017015) coupled with Cell Sense software (Olympus, RRID:SCR_016238).

### 4.8. Statistical Analysis

Prism 5.0 (Graphpad Software, La Jolla, CA, USA, RRID:SCR_002798) statistical software was used to analyse the results. The non-parametric Mann–Whitney *U* test (for unpaired observations) was used to compare groups of data. The associations between the clinicopathological parameters and the expression of the IHC markers were analysed using the Chi^2^ test. The survival times were determined by the Kaplan–Meier method, and the significance of the differences was assessed by the log-rank test. For each variable, the hazard ratio (HR) and the 95% confidence interval (95% CI) were estimated. In all the analyses, the results were considered statistically significant at *p* < 0.05.

## 5. Conclusions

This study has been the first to show on a large and homogenous population of patients a significantly increased level of POSTN expression in CAFs in NSCLC and in two histological subtypes (SCC and AC) compared to NMLT. We demonstrated focal colocalisation between POSTN and CAF-associated markers (α-SMA and D2-40), which confirms that CAFs are an important source of POSTN in NSCLC. Moreover, the IHC analysis of POSTN showed significant relationships between the glycoprotein and the clinicopathological data of patients (e.g., clinical cancer stage, tumour size, or lymph node involvement). Our results showed that POSTN-positive CAFs can be an independent negative prognostic factor for survival in patients with NSCLC as well as in individual histological subtypes (SCC and AC).

## Figures and Tables

**Table 1:** Survival analysis of (**A**) the whole cohort and (**B**) squamous cell cancer (SCC) and adenocarcinoma (AC). Cox proportional hazards regression.

**A**								
**Overall Survival (OS)**								
**Clinicopathological Parameters**	**NSCLC**							
	**Univariate Analysis**				**Multivariate Analysis**			
	***p*** **-Value**		**HR (95% HR CI)**		***p*** **-Value**		**HR (95% HR CI)**	
Age (<62 vs. >62)	**0.0002**		1.39 (1.17–1.66)		**0.0001**		1.41 (1.18–1.69)	
POSTN CAFs (low vs. high)	**<0.0001**		1.15 (1.12–1.18)		**<0.0001**		1.12 (1.09–1.15)	
Ki-67 (median)	0.11		1.15 (0.97–1.37)		-		-	
Chronic obstructive pulmonary disease	0.08		1.17 (0.98–1.39)		-		-	
Hypertension	0.57		1.05 (0.88–1.25)		-		-	
Coronary artery disease	**0.02**		1.60 (1.07–2.39)		**0.02**		1.64 (1.09–2.45)	
Smoking history	0.29		1.01 (0.99–1.03)		-		-	
Stage (I vs. II-IV)	**<0.0001**		2.21 (1.84–2.64)		**0.01**		1.42 (1.10–1.84)	
Grade (G1 vs. G2-G3)	0.49		0.88 (0.61–1.27)		-		-	
Tumour size (T1–T2 vs. T3–T4)	**<0.0001**		1.92 (1.60–2.29)		**0.0008**		1.44 (1.16–1.79)	
Lymph nodes involvement (N0 vs. N+)	**<0.0001**		1.84 (1.54–2.19)		**0.0005**		1.48 (1.19–1.85)	
pM	0.12		1.89 (0.84–4.23)		-		-	
**B**								
**Overall Survival (OS)**								
**Clinicopathological Parameters**	**SCC**				**AC**			
	**Univariate Analysis**		**Multivariate Analysis**		**Univariate Analysis**		**Multivariate Analysis**	
	***p*** **-Value**	**HR (95% HR CI)**	***p*** **-Value**	**HR (95% HR CI)**	***p*** **-Value**	**HR (95% HR CI)**	***p*** **-Value**	**HR (95% HR CI)**
Age (<62 vs. >62)	**0.05**	1.32 (1.01–1.73)	**0.03**	1.35 (1.02–1.79)	**0.03**	1.36 (1.03–1.79)	**0.003**	1.53 (1.15–2.02)
POSTN CAFs (low vs. high)	**0.0007**	1.62 (1.23–2.14)	**0.001**	1.58 (1.20–3.28)	**<0.0001**	1.22 (1.16–1.27)	**<0.0001**	1.21 (1.15–1.27)
Ki-67 (median)	0.53	0.92 (0.70–1.20)	-	-	**0.04**	1.37 (1.02–1.83)	0.21	-
Chronic obstructive pulmonary disease	0.44	1.11 (0.85–1.46)	-	-	0.23	1.18 (0.90–1.56)	-	-
Hypertension	0.73	1.05 (0.81–1.37)	-	-	0.33	0.87 (0.66–1.15)	-	-
Coronary artery disease	0.73	1.05 (0.81–1.37)	-	-	0.13	1.88 (0.83–4.24)	-	-
Smoking history	0.37	1.01 (0.99–1.03)	-	-	**0.03**	1.52 (1.04–2.23)	0.12	1.36 (0.92–2.01)
Stage (I vs. II-IV)	**<0.0001**	1.83 (1.38–2.44)	0.13	1.38 (0.91–2.09)	**<0.0001**	2.44 (1.84–3.23)	0.43	1.18 (0.78–1.78)
Grade (G1 vs. G2-G3)	0.31	1.48 (0.70–3.14)	-	-	0.15	0.71 (0.45–1.13)	-	-
Tumour size (T1-T2 vs. T3-T4)	**0.0001**	1.75 (1.33–2.31)	**0.02**	1.50 (1.07–2.11)	**<0.0001**	2.16 (1.62–2.88)	**0.01**	1.56 (1.11–2.19)
Lymph nodes involvement (N0 vs. N+)	**0.07**	1.47 (1.11–1.93)	0.16	1.28 (0.91–1.81)	**<0.0001**	2.25 (1.70–2.98)	**0.0001**	2.04 (1.41–2.95)
pM	**0.03**	9.4405 (1.29–68,82)	0.12	5.01 (0.67–3.68)	0.88	1.09 (0.35–3.42)	-	-

Significant *p*-values are given in bold. HR—hazard ratio; CI—confidence interval; POSTN—periostin; NSCLC—non-small cell lung cancer; SCC—squamous cell carcinoma; AC—adenocarcinoma.

**Table 2:** Relationship between periostin (POSTN) expression in the stromal cells (CAFs) and selected clinicopathological parameters in non-small cell lung cancer (NSCLC) patients and in two histological subtypes: adenocarcinoma (AC) and squamous cell cancer (SCC).

Characteristics	NSCLC (*N* = 700)			AC (*N* = 330)			SCC (*N* = 370)		
	POSTN Expression by CAFs			POSTN Expression by CAFs			POSTN Expression by CAFs		
	Low	High	Chi^2^ Test *p*-Value	Low	High	*p*-Value	Low	High	Chi^2^ Test *p*-Value
	*n* (%)	*n* (%)		*n* (%)	*n* (%)		*n* (%)	*n* (%)	
Age									
≤63	182 (26%)	194 (27.7%)	0.76	101 (30.6%)	89 (27%)	0.33	81 (21.9%)	105 (28.4%)	0.99
>63	158 (22.6%)	166 (23.7%)		78 (23.6%)	62 (18.8%)		80 (21.6%)	104 (28.1%)	
Tumour size									
pT1	97 (13.9%)	75 (10.7%)	**0.03**	52 (15.8%)	27 (8.2%)	**0.03**	45 (12.2%)	48 (13%)	0.27
pT2–pT4	243 (34.7%)	285 (40.7%)		127 (38.5%)	124 (37.6%)		116 (31.4%)	161 (43.6%)	
Grade									
G1	37 (5.3%)	5 (0.7%)	**<0.0001**	31 (9.4%)	1 (0.3%)	**<0.0001**	6 (1.6%)	6 (1.6%)	0.15
G2	247 (35.3%)	271 (38.7%)		111 (33.6%)	106 (32.1%)		136 (36.8%)	165 (44.6%)	
G3	53 (7.6%)	87 (12.4%)		35 (10.6%)	46 (13.9%)		18 (4.9%)	39 (10.5%)	
Lymph node involvement									
pN0	238 (34%)	211 (30.1%)	**0.005**	126 (38.2%)	83 (25.2%)	**0.02**	112 (30.3%)	128 (34.6%)	0.1
pN1, N2	102 (14.6%)	149 (21.3%)		53 (16.1%)	68 (20.6%)		49 (13.2%)	81 (21.9%)	
Stage									
I	149 (21.3%)	105 (15%)	**0.0001**	83 (25.2%)	40 (12.1%)	**0.0006**	65 (17.6%)	66 (17.8%)	0.1
II-IV	191 (27.3%)	255 (36.4%)		96 (29.1%)	111 (33.6%)		95 (25.7%)	144 (38.9%)	

Significant *p*-values are given in bold. NSCLC—non-small cell lung cancer; AC—adenocarcinoma; SCC—squamous cell carcinoma.
